# Clinical efficacy and safety of switching from eculizumab to ravulizumab in adult patients with aHUS– real-world data

**DOI:** 10.1186/s12882-024-03638-3

**Published:** 2024-06-19

**Authors:** Kristina Schönfelder, Lucas Kühne, Lena Schulte-Kemna, Jessica Kaufeld, Hana Rohn, Andreas Kribben, Bernd Schröppel, Paul T. Brinkkötter, Anja Gäckler

**Affiliations:** 1 Department of Nephrology, University Hospital Essen, University Duisburg-Essen, Essen, Germany; 2 Department II of Internal Medicine and Center for Molecular Medicine Cologne, University Hospital Cologne, University of Cologne, Cologne, Germany; 3 Section of Nephrology, Ulm University Hospital, University of Ulm, Ulm, Germany; 4 Division of Nephrology, Center for Internal Medicine, Hannover Medical School, Hannover, Germany; 5 Department of Infectious Diseases, University Hospital Essen, University Duisburg-Essen, Essen, Germany; 6 Klinik Für Nephrologie, Universitätsklinikum Essen, Hufelandstr. 55, Essen, 45147 Germany

**Keywords:** Atypical hemolytic uremic syndrome, Thrombotic microangiopathy, Eculizumab, Ravulizumab, Kidney transplant recipients

## Abstract

**Background:**

The complement factor 5 (C5)-inhibitor eculizumab has been established as standard-of-care for the treatment of atypical hemolytic uremic syndrome (aHUS). In 2021, the long-acting C5-inhibitor ravulizumab was approved, extending intervals of intravenous treatment from two to eight weeks resulting in improvement of quality of life for patients and lowering direct and indirect therapy associated costs.

**Methods:**

This multicenter, retrospective data analysis of 32 adult patients with aHUS (including 10 kidney transplant recipients) treated with eculizumab for at least three months and switched to ravulizumab aims to evaluate the safety and efficacy of switching medication in the real-world setting. Hematologic parameters, kidney function, concurrent therapy and aHUS associated events were evaluated three months before and until up to 12 months after switching to ravulizumab.

**Results:**

Mean age (range) at ravulizumab initiation was 41 years (19–78 years) and 59% of the patients were female. Genetic analysis was available for all patients with 72% showing a pathogenic variant. Median time (range) on eculizumab before switching was 20 months (3–120 months). No new events of TMA or worsening of renal function were reported during up to 12 months of follow-up during ravulizumab treatment.

**Conclusions:**

This is the largest, non-industry derived, multi-center retrospective analysis of adult patients with aHUS switching C5-inhibitor treatment from eculizumab to ravulizumab in the real-world setting. Switching to ravulizumab was safe and efficient resulting in sustained hematological stability and preservation of renal function.

**Supplementary Information:**

The online version contains supplementary material available at 10.1186/s12882-024-03638-3.

## Background

Atypical hemolytic uremic syndrome (aHUS) is a rare, but life-threatening disease with dysregulated complement activity leading to thrombotic microangiopathy (TMA) resulting in thrombocytopenia, hemolytic anemia, and multi-organ dysfunction commonly including kidney impairment [1]. Before the establishment of complement factor 5 (C5)-inhibition as standard of care, therapy was limited to plasma exchange with poor clinical outcome. Within 5 years, about 2/3 of adult patients progressed to end-stage renal disease or death [2, 3]. The approval of the monoclonal C5 inhibitor eculizumab for the treatment of aHUS in 2011 was game changing. Eculizumab administered intravenously every two weeks following a weekly induction is able to normalize hematologic parameters and to significantly improve kidney function and thereby reduce short-term and long-term need for renal replacement therapy [4]. Eculizumab has also proved to be beneficial in aHUS in the context of renal transplantation [5].

The long-acting C5-inhibitor ravulizumab was developed from eculizumab and was approved for treatment of aHUS in Europe in 2021. A change in four amino acids increases the affinity of the monoclonal antibody for the neonatal Fc receptor and enhances pH dependent antibody recycling thereby extending duration of action [6, 7]. Thus, with the same epitope, a similar affinity and rate ravulizumab has a half-life period of approximately 52 days in contrast to eculizumab with 11 days [8] allowing for dosing intervals of 8 weeks following induction. In the phase III trial evaluating the efficacy and safety of ravulizumab in adult patients with aHUS complete TMA response was achieved in 54% of the patients and 59% of the patients on dialysis at baseline came off dialysis within 6 months. The trial also included renal transplant recipients (14.3%) [8]. Treatment with ravulizumab resulted in immediate, complete, and sustained terminal complement inhibition as defined by free C5 in serum concentrations less than 0.5 µg/ml [8]. Efficacy and safety was confirmed during long-term follow up (median follow-up 76.7 weeks) [9]. Although, a comparative trial of eculizumab versus ravulizumab was not performed, indirect comparison using clinical trial data did not reveal any difference in efficacy or safety [10]. Data from clinical trials of ravulizumab treatment in myasthenia gravis using the identical dosing regimen as in aHUS showed complete post-dose inhibition indicated by free C5 concentrations below the regulatory-accepted threshold of 0.5 µg/ml, being superior to eculizumab with 92% complete inhibition at all time points [11–13].

Due to reduction of direct and indirect treatment costs and increased quality of life associated with lower treatment burden switching from eculizumab to ravulizumab is routinely performed in patients with long-term treatment indications. No study in adult patients with aHUS showing that a switch in medication from eculizumab to ravulizumab is efficient and safe has been reported. Therefore, we initiated a multi-center, retrospective analysis of adult patients with controlled aHUS who were switched from eculizumab to ravulizumab.

## Methods

This multicenter retrospective analysis was designed to evaluate the clinical safety and efficacy of switching medication from eculizumab to ravulizumab in adult patients with aHUS in a real-world setting. All patients had a clinical diagnosis of aHUS and were successfully treated with eculizumab for at least three months before switching to ravulizumab as demanded by the current drug approval [14]. Complement inhibitor treatment naïve patients with a de novo diagnosis of aHUS were not included in the study. Patients with a follow up time of at least six months were included in the study. All patients were older than 18 years. The study was approved by the Ethic Committee of the University of Duisburg-Essen (23–11,248-BO) as well as by the Ethic Committees of the Medical School Hannover, and the Universities of Cologne and Ulm.

Ravulizumab (Ultomiris®) loading dose was administered intravenously two weeks after the last eculizumab (Soliris®) infusion. The second ravulizumab infusion was administered two weeks thereafter and then every eight weeks intravenously. Dose was adjusted to body weight as recommended in the prescribing information [14].

All patients presented with stable disease under eculizumab treatment. Hematological and renal parameters were analyzed approximately three months before starting ravulizumab treatment, the day of switching from eculizumab to ravulizumab, and approximately three, six and twelve months after switching. No patient terminated treatment with ravulizumab during the follow up. All patients had an updated vaccination status against meningococci.

Primary efficacy endpoint was a stable disease monitored by hematological parameters (platelet count, hemoglobin, lactate dehydrogenase (LDH) level and haptoglobin) and a conserved kidney function measured via serum creatinine. Furthermore, safety parameters as hospitalization, treatment related side effects or other adverse outcomes were ascertained during clinical visits.

Neither pharmacokinetic or pharmacodynamic information on eculizumab or ravulizumab, nor data on free C5 concentration or complement status (e.g. CH50, AH50 or soluble C5b-9) were available in this retrospective, real-world analysis.

For subgroup analysis patients were stratified by renal status (non-transplant vs. renal transplant) and time since diagnosis of aHUS (> 6 vs. < 6 months).

Data were analyzed using GraphPadPrism 8.4.2.679 (San Diego, CA, USA). Differences over time or between subgroups were analyzed by mixed-effects analysis. A p-value < 0.05 was considered significant.

## Results

Thirty-two patients were included in the study. Eculizumab was administered for a duration of 3–120 months (mean 28.7 ± 28.7 months, median 20 months) before the first dose of ravulizumab.

Mean age of 41.4 ± 16 years and 40.6% were male. 50% of the patients presented with three or more comorbidities. The most common comorbidities were arterial hypertension, followed by coronary artery disease and hypothyroidism.

Ten patients (31%) had previously received a kidney transplant. Renal transplant recipients showed more comorbidities than non-transplanted patients.

Time from last transplantation until the treatment with ravulizumab was 46.4 months (range 2—158). Six patients received a kidney transplant because of end-stage renal disease due to aHUS. Other reasons for kidney transplantation were Alport syndrome, polycystic kidney disease, IgA nephropathy and renal dysplasia in combination with aHUS. Four patients required more than one kidney transplantation. One patient (primary disease: aHUS) lost her previous transplant due to chronic rejection. The other three patients lost their transplants due to genetically proven aHUS. Four had received a living kidney donation. Immunosuppressive regimes included steroids in combination with tacrolimus and/or mycophenolate mofetil or everolimus.

Genetic testing was available in all patients. 71.9% presented with at least one known pathogenic variant in the complement system associated with aHUS. The majority (74%) of pathogenic variants found was in complement factor H.

During the three months before switching to ravulizumab patients either presented with a stable disease under eculizumab treatment or showed therapeutic benefit when C5 inhibition was initiated shortly before. Patient characteristics are shown in Table 1.

**Table 1:** Patient characteristics

	All (*n* = 32)	Non-transplanted patients (*n* = 22)	Renal transplant recipients (*n* = 10)
Sex			
Male % (n)	40.6 (13)	40.9 (9)	40.0 (4)
Age			
at first ravulizumab infusion in years (range)	41.4 (19–78)	42.9 (19–78)	38.1 (20–72)
at first occurrence of aHUS in years (range)	34.5 (3–73)	38.6 (3–73)	24.2 (6–42)^a^
Comorbidities % (n)			
0	21.9 (7)	27.3 (6)	10.0 (1)
1	18.8 (6)	22.7 (5)	10.0 (1)
2	9.4 (3)	4.5 (1)	20.0 (2)
3	18.8 (6)	18.2 (4)	20.0 (2)
> 3	31.2 (10)	27.3 (6)	40.0 (4)
Duration of eculizumab treatment in months (range)	28.7 (3–120)	30.9 (3–120)	22.9 (3–51)^b^
Patients with ≥ 1 pathogenic genetic variant % (n)	71.9 (23)	77.3 (17)	60.0 (6)
Time since complement mutation analysis			
≥ 1 pathogenic genetic variant; years (range)	5.7 (0–15)	4.6 (0–11)	8.8 (2–15)
no pathogenic genetic variant; years (range)	5.6 (1–12)	4.5 (1–8)	6.8 (1–12)

^a^Data missing for one patient with a highly positive family history and diagnosis > 10 years ago

^b^For two renal transplant recipients, duration of eculizumab was not known

Supplementary Table 1 offers more precise information on genetic mutations, age at diagnosis of aHUS and treatment duration with eculizumab.

There were 15 adverse events (Table 2). The only hospitalization was for kidney biopsy in one patient not being related to C5 inhibitor treatment.

**Table 2:** Adverse events after switch to ravulizumab

	% of adverse events	number of patients
**Serious adverse event**^a^		1
**Adverse event**		18
Upper respiratory tract infection	22.2	4
Headache/dizziness	22.2	4
Cutaneous infusion reaction	16.7	3
Transaminase elevation	5.6	1
Edema	5.6	1
Urinary tract infection	27.8	5
Meningococcal infection/death	0	0

^a^Serious adverse event was hospitalization for kidney biopsy rated as not-associated to C5 inhibitor treatment. Urinary tract infections were reported in renal transplant recipients only

No clinical signs of TMA relapse were reported during the study period.

**Fig. 1:**
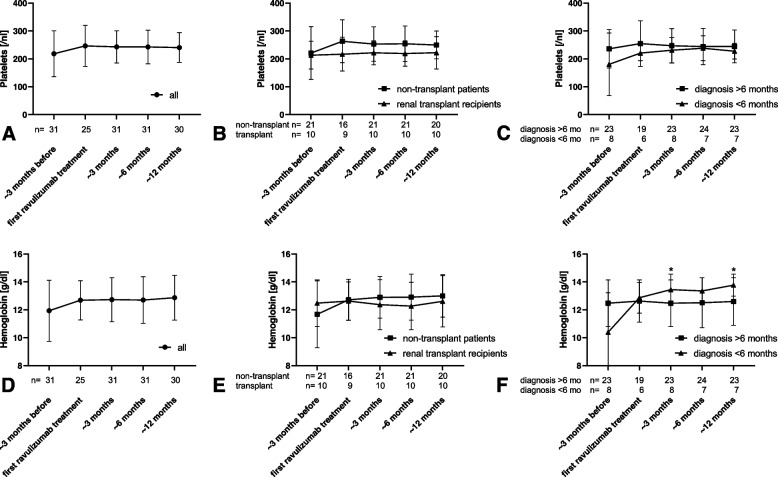
Platelets and hemoglobin. Platelets (**A**-**C**) and hemoglobin (**D**-**F**) approx. 3 months before and up to approx. 12 months after switch from eculizumab to ravulizumab. *A* + *D* all patients; *B* + *E* non-transplanted patients and renal transplant recipients; *C* + *F* patients with diagnosis of aHUS > 6 months and < 6 months before switch of medication. * p < 0.05 vs. approx. 3 months before switching to ravulizumab

**Fig. 2:**
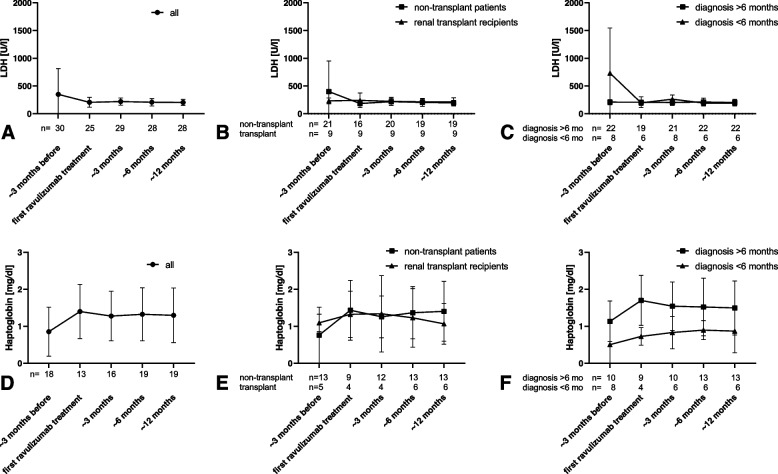
LDH and haptoglobin. LDH (**A**-**C**) and haptoglobin (**D**-**F**) approx. 3 months before and up to approx. 12 months after switch from eculizumab to ravulizumab. *A* + *D* all patients; *B* + *E* non-transplanted patients and renal transplant recipients; *C* + *F* patients with diagnosis of aHUS > 6 months and < 6 months before switch of medication

No significant changes in the hematologic parameters such as platelet count (Fig. 1 A-C), hemoglobin (Fig. 1 D-F), LDH (Fig. 2 A-C) or haptoglobin (Fig. 2 D-F) were measured during the study period regarding all patients.

No differences were detected between non-transplanted patients and renal transplant recipients. Hematological parameters improved in patients with diagnosis of aHUS less than six months before switching to ravulizumab compared to values three months before the first ravulizumab infusion reaching significance for hemoglobin at 3 and 12 months (Fig. 1 E, *p*< 0.05).

**Fig. 3:**
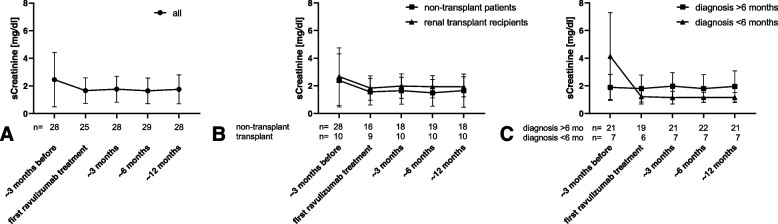
Serum creatinine. Serum creatinine approx. 3 months before and up to approx. 12 months after switch from eculizumab to ravulizumab. **A** all patients; **B** non-transplanted patients and renal transplant recipients; **C** patients with diagnosis of aHUS > 6 months and < 6 months before switch of medication

Serum creatinine remained stable in all patients throughout the study (Fig. 3). Three non-transplanted patients remained dependent on renal replacement therapy throughout the study and were therefore excluded from analysis of serum creatinine. Seven patients with diagnosis less than six months before switching of medication were available for analysis. Serum creatinine decreased from 4.1 ± 3.2 mg/dl to 1.2 ± 0.4 mg/dl in those patients, not reaching statistical significance. No patient progressed to end stage renal disease within the study period.

## Discussion

This retrospective multicenter study demonstrates the clinical efficacy and safety of switching C5 inhibitor treatment from eculizumab to ravulizumab in adult patients with aHUS in a real-world setting.

All patients included in the study had undergone genetic analysis for complement mutations. A pathogenic variant was confirmed in 71.9%. This proportion is in line with literature in which pathogenic variants are found in approximately 60–70% [2, 15], but is higher than in the ravulizumab phase 3 trial and the global aHUS registry in which only 20.5% and 45% respectively had a genetically confirmed diagnosis [8, 16]. However, genetic testing was performed in a smaller proportion of those cohorts, but is regularly performed as routine work-up of aHUS patients in Germany. Complement mutation analysis, respectively interpretation of such should be repeated periodically, especially in patients with variants of unknown significance or initial negative results. Classification of complement gene variants is a continuously evolving process and interpretation of variants can change over time [17]. While genetic results are not required for diagnosis, they can potentially enable individualized treatment approaches.

The concept of C5 inhibition in patients with aHUS has evolved from life-long treatment to individualized concepts. As aHUS is a rare disease study data is limited, but individual risk for relapse after discontinuation of C5 inhibition seems to vary between < 25–50% [18, 19]. Different algorithms can be used to assess the risk for recurrence [18, 20, 21]. However, a treatment duration of at least 6 to 12 months is commonly recommended [22]. Besides occurrence in early childhood, relapses, persistent complement activity and identification of triggers, underlying genetic mutations and transplant status are important components for evaluation of an appropriate treatment duration. The authors of this manuscript are well aware that prolonged treatment is not needed in all patients with aHUS. This is also reflected by the fact that all patients included in this study had undergone genetic testing. However, necessity for C5 inhibition was determined for all patients included in the study by the treating physicians at the time of the study.

Adverse events were reported by 47% of the patients while treated with ravulizumab. Most events were rated as not being associated with complement inhibitor treatment. Treatment related adverse events such as cutaneous infusion reactions were in line with those reported in clinical trials [8, 23]. Hepatotoxicity has neither been reported for eculizumab nor ravulizumab during clinical trials. Nevertheless, hepatotoxicity of eculizumab has been observed in children and adults independent of the treated disease [24–26]. As to our knowledge, reports on clinically apparent liver injury associated with the use of ravulizumab have not been reported. Elevation of transaminases reported for one patient of our cohort was mild, transient and resolved without further actions.

Importantly, none of the AEs resulted in treatment discontinuation.

C5 inhibition significantly increases the risk of meningococcal infections with no difference between substances [27]. We did not observe any meningococcal infection in our study. Antibiotic prophylaxis against meningococcal infections is required from the time of first dose of C5 inhibition until at least 2 weeks after vaccination [14]. It is a common approach, that patients are equipped with stand-by antibiotics in order to minimize duration to treatment in case of suspected meningococcal infection [28]. Besides a growing cumulative exposure to C5 inhibitors due to extended approval for different diseases, numbers of meningococcal infection rates have steadily decreased and mortality rates remained low [29].

Efficacy and safety of ravulizumab have been demonstrated for adult patients with de novo initiation of C5 inhibitor treatment in aHUS [8]. In clinical studies measurement of free C5 in serum was used to confirm complement inhibition. Unfortunately, testing of free C5 is not commercially available for clinical use. In addition and in contrast to eculizumab, complement blockade by ravulizumab cannot be assessed using CH50 [30, 31]. It is hypothesized that with use of ravulizumab, there could be release of C5 and inaccurate readings of results, since some of the assays use low pH in vitro [31]. It is suggested, that measurement of AH50 in combination with ravulizumab concentration measurement may provide an opportunity for adequate therapeutic drug monitoring [30]. However, measurement of complement assays or drug concentrations has not been validated in a broader clinical setting and is rarely performed in routine clinical practice. Due to the retrospective character of our real-world study and as a relevant limitation, data on pharmacokinetics and pharmacodynamics, as well as on free C5 concentration or complement status (e.g. CH50, AH50 or soluble C5b-9) are not available.

In addition, switching medication from eculizumab to ravulizumab resulted in stable hematological and renal parameters without unexpected safety concerns in children with aHUS [23]. Switching has also been shown to be feasible in adult patients following renal transplantation [32]. Multinational registry data presented at the American Society of Nephrology Kidney Week 2023 including 60 patients (24 pediatric) were in line with our results showing sustained maintenance of kidney function without evidence of new events of dialysis, kidney transplant, or TMA relapse [33].

Reduction of dosing frequency is the main advantage of switching from eculizumab to ravulizumab improving quality of life in adult and pediatric patients with aHUS. Patients spend less time within health care facilities generally resulting in better adherence to treatment and increased compliance [34]. Lower medication costs, also in relation to the eculizumab biosimilar which has recently obtained an indication extension for aHUS in the European Union [35], and resultant productivity implications for patients with aHUS and their caregivers even make switching from eculizumab to ravulizumab cost effective for long-term treatment [36].

Data are limited by the retrospective character of the study, the missing information on pharmacokinetics/pharmacodynamics and complement assays and we were unable to assess quality of life.

## Conclusions

This is the largest, non-industry derived, multi-center retrospective analysis of adult patients with aHUS switching C5-inhibitor treatment from eculizumab to ravulizumab in the real-world setting. Switching to ravulizumab was safe and efficient resulting in sustained hematological stability and preservation of renal function.

### Supplementary Information

Supplementary Material 1.

## Data Availability

The datasets used and/or analysed during the current study are available from the corresponding author on reasonable request.

## Abbreviations

aHUS: Atypical hemolytic uremic syndrome
TMA: Thrombotic microangiopathy
C5: Complement factor 5
LDH: Lactate dehydrogenase
