# Supplementary material for: Clinical efficacy and safety of switching from eculizumab to ravulizumab in adult patients with aHUS– real-world data
Source: BMC Nephrol. 2024 Jun 19;25:202. doi: 10.1186/s12882-024-03638-3 (PMC11188157; doi:10.1186/s12882-024-03638-3)
Supplement: Supplementary file 1 — Supplementary Material 1. [file 12882_2024_3638_MOESM1_ESM.docx]

**Supplementary Table 1. Genetic mutations, age at diagnosis, and treatment duration with eculizumab**

| **Mutation** | **Year of genetic mutation analysis** | **Age at diagnosis [years]** | **Treatment duration with eculizumab [months]** | **Renal disease** | **Previous transplants** |  |
| --- | --- | --- | --- | --- | --- | --- |
| ***Non-transplant patients*** | | | | | | |
| C3 | 2018 | 3 | 33 |  |  |  |
| CFH, CFHR3/R1 + Anti-CFH antibodies | 2012 | 10 | 120 |  |  |  |
| CD46 | 2022 | 21 | 3 |  |  |  |
| MCP | 2016 | 22 | 53 |  |  |  |
| CFH | 2021 | 22 | 7 |  |  |  |
| CFH | 2021 | 23 | 4 |  |  |  |
| CFI | 2019 | 24 | 19 |  |  |  |
| CFI | 2013 | 32 | 96 |  |  |  |
| CFI | 2020 | 32 | 16 |  |  |  |
| CFH | 2023 | 32 | 3 |  |  |  |
| - | 2016 | 38 | 28 |  |  |  |
| CFB, CFH, CFI | 2015 | 40 | 66 |  |  |  |
| CFH | 2018 | 48 | 15 |  |  |  |
| CFH | 2015 | 49 | 32 |  |  |  |
| - | 2017 | 51 | 37 |  |  |  |
| CFH | 2021 | 52 | 4 |  |  |  |
| CFH | 2023 | 52 | 3 |  |  |  |
| - | 2022 | 53 | 5 |  |  |  |
| - | 2021 | 56 | 4 |  |  |  |
| - | 2015 | 56 | 60 |  |  |  |
| CFHR1/CFH | 2019 | 61 | 20 |  |  |  |
| positive (not specified) | Not specified | 73 | 51 |  |  |  |
| **Renal transplant recipients** | | | | | | |
| C3/4 | 2010 | 6 | Not specified | Atypical hemolytic uremic syndrome | 1 |  |
| CFH, CFHR3/R1 | 2014 | 15 | 11 | Atypical hemolytic uremic syndrome | 1 |  |
| - | 2011 | 16 | 29 | Atypical hemolytic uremic syndrome | 1 |  |
| - | 2015 | 19 | Not specified | Atypical hemolytic uremic syndrome |  |  |
| - | 2022 | 19 | 3 | Renal dysplasia, Atypical hemolytic uremic syndrome |  |  |
| Deletions in CFHR1, CFHR3, CFHR4 | 2021 | 26 | 5 | Autosomal-dominant polycystic kidney disease |  |  |
| - | 2017 | 36 | 51 | IgA nephropathy |  |  |
| CHF, CFI | 2008 | 39 | 35 | Atypical hemolytic uremic syndrome |  |  |
| CFH | 2017 | 42 | 5 | Alport syndrome, Atypical hemolytic uremic syndrome | 2 |  |
| MCP | 2015 | Not specified | 44 | Atypical hemolytic uremic syndrome |  |  |
